# The Predictive Accuracy of Anogenital Distance and Genital Tubercle Angle for First-Trimester Fetal Sex Determination

**DOI:** 10.3390/diagnostics14161811

**Published:** 2024-08-20

**Authors:** Abdulrahman M. Alfuraih, Bashaier Mansour Almajem, Amal Abdullah Alsolai

**Affiliations:** 1 Radiology and Medical Imaging Department, College of Applied Medical Sciences, Prince Sattam bin Abdulaziz University, Kharj 11942, Saudi Arabia; 2 Obstetrics and Gynecology Ultrasound Department, Prince Sultan Military Medical City, Riyadh 11159, Saudi Arabia; balmajem@psmmc.med.sa; 3 Department of Radiological Sciences, College of Applied Medical Sciences, King Saud University, Riyadh 11421, Saudi Arabia; amalsolai@ksu.edu.sa

**Keywords:** fetal gender determination, anogenital distance, genital tubercle angle, first-trimester ultrasound, prenatal diagnosis

## Abstract

Background: Early identification of fetal gender is crucial for managing gender-linked genetic disorders. This study aimed to evaluate the predictive performance of anogenital distance (AGD) and genital tubercle angle (GTA) for fetal sex determination during the first trimester. Methods: A multicenter retrospective cohort study was conducted on 312 fetal cases between 11 and 13 + 6 weeks of gestation from two tertiary hospitals. AGD and GTA measurements were taken from midsagittal plane images using ultrasound, with intra- and inter-reader reproducibility assessed. Binomial logistic regression and ROC curve analysis were employed to determine the diagnostic performance and optimal cutoff points. Results: AGD had a mean of 7.16 mm in male fetuses and 4.42 mm in female fetuses, with a sensitivity of 88.8%, specificity of 94.4%, and an area under the ROC curve (AUC) of 0.931 (95% CI: 0.899–0.962) using 5.74 mm as a cutoff point. For GTA, the mean was 35.90 degrees in males and 21.57 degrees in females, with a sensitivity of 92%, specificity of 84.7%, and an AUC of 0.932 (95% CI: 0.904–0.961) using 28.32 degrees as a cutoff point. The reproducibility results were excellent for AGD (intra-operator ICC = 0.938, inter-operator ICC = 0.871) and moderate for GTA (intra-operator ICC = 0.895, inter-operator ICC = 0.695). Conclusions: The findings suggest that AGD and GTA are reliable markers for early fetal sex determination, with AGD showing higher reproducibility. The findings highlight the feasibility and accuracy of these non-invasive sonographic markers and their potential usefulness in guiding timely interventions and enhancing the management of gender-linked genetic conditions.

## 1. Introduction

Beyond parents’ curiosity, early identification is crucial, particularly for fetuses at high risk of inheriting gender-linked genetic disorders. Male fetuses can be affected by X-linked recessive diseases, such as hemophilia and Duchenne muscle dystrophy. Conversely, female fetuses may suffer from conditions like adrenal congenital hyperplasia, which can lead to virilization and life-threatening complications if not managed promptly. Early and accurate determination of fetal gender allows for timely diagnosis and interventions, ensuring that appropriate treatments can be initiated as soon as possible. 

This early identification also aids in planning prenatal diagnostic procedures. It can guide decisions regarding invasive diagnostic tests like chorionic villus sampling or amniocentesis [1]. These tests can confirm the presence of specific genetic disorders, enabling informed decision-making and preparation for postnatal management. Hence, accurate identification of fetal gender in early pregnancy is not just a matter of interest but a critical component of prenatal care, aiding in the management of gender-specific genetic disorders.

Early sex determination can be achieved using genetic testing. Specifically, chorionic villus sampling under ultrasound guidance provides definitive results but is invasive and carries a relative risk of pregnancy loss before 28 weeks [2]. Alternatively, non-invasive prenatal testing through cell-free fetal DNA offers a non-invasive option; however, it is expensive and limited in availability [3,4,5,6,7]. 

The earliest sonographic sex determination attempts started in the late 1970s [8,9,10]. To our knowledge, Stocker et al., in 1997, were the first to describe the sonographic technique and accuracy rates of sonographic sex determination [8]. Before the advent of modern high-frequency and resolution US imaging, scanning feasibility was the main challenge. Most attempts were unsuccessful, with high rates (54–63%) of indeterminate results [9,10,11,12]. However, when the genitalia were able to be visualized, the accuracy results were close to 100%. In such early studies, sex determination was based on the morphological characteristics of external genitalia (labia and scrotum) in addition to the internal genital organs in the pelvic floor, such as the uterus, ovaries, and testes [13]. All attempts were limited to pregnancies of 30 weeks of gestation and older, as first-trimester sex determination was not possible due to the poor spatial resolution in such old systems and the lack of novel sonographic markers that will be reviewed next. Furthermore, the penis and clitoris demonstrate no appreciable differences in size until after 14 weeks of gestation, delaying any early trimester attempts using such conventional techniques.

Later, the methods of genital tubercle angle (GTA) and ‘sagittal sign’ were introduced as methods of sex determination during the first trimester [14,15,16]. They focus on the direction of the genital tubercle relative to the lumbosacral surface, where a high angle reflecting an upward genital tubercle direction reflects a male fetus [14,15]. It soon became the method of choice for sex determination in early pregnancy, starting from 11 weeks and higher [16]. 

A novel sonographic imaging marker, anogenital distance (AGD), has received growing interest [17,18,19,20,21,22,23,24]. AGD measures the distance from the anus to the genitalia, showing sexual dimorphism, with males having longer AGD than females. It was initially used in rodents to distinguish between sexes. AGD is determined within a critical window of androgen action, around 10 weeks gestation, when sex differentiation begins, developing toward male phenotypes under the influence of androgen. At birth, AGD serves as a non-invasive indicator of androgen levels and can predict abnormal reproductive system development [25,26,27]. 

Despite the rising interest in AGD, reference data remain scarce. Moreover, no previous studies have compared AGD against GTA in a single cohort of first-trimester pregnancies. This study was designed to address the need for reliable, non-invasive methods for early fetal gender determination, which is essential for managing gender-linked genetic disorders. While existing literature has explored genetic testing methods, these are either invasive or costly. By evaluating the fetal sex predictive performance of AGD and GTA, this study not only fills a gap in the literature but also investigates if AGD and GTA could be feasible and practical tools in early prenatal care. Hence, the primary objective of this study was to investigate the fetal sex predictive performance of AGD and GTA during the first trimester. A secondary objective was to assess the intra- and inter-reader reproducibility of the sonographic markers.

## 2. Materials and Methods

### 2.1. Study Design and Participants

The study was designed as a multicenter retrospective cohort study at Prince Sultan Military Medical City and King Faisal Specialist Hospital. The data were collected from January 2020 to January 2022. The local research ethics committee granted a consent waiver and approval (No: 1575). Hospital records were reviewed for obstetric scans meeting the eligibility criteria, which included singleton pregnancies with a gestational age between 11 and 13 + 6 weeks and images acquired transabdominally in the midsagittal plane of the fetus. Additionally, only Saudi nationals were included to avoid potential racial confounding factors, as reported in previous studies [19,20,23]. Exclusion criteria were cases where the fetus had a hyperflexed or hyperextended neck or spine or if the gender at birth was not documented. To mitigate the potential bias of retrospective cohort designs, the selection of cases was based on the availability of ultrasound images that met the inclusion criteria, thereby minimizing selection bias. Furthermore, collecting the data from two different hospitals provided a broader population base, further enhancing the generalizability of the findings and reducing the potential for bias.

### 2.2. Data Collection

Drawing from previously published data [24], a sample size of at least 250 subjects is necessary to detect a minimum 10% difference in AGD using an independent sample t-test. This calculation is based on an alpha level of 0.05 and a statistical power (1 − β) of 0.95, assuming an equal distribution between male and female groups. An accredited sonographer (BA) with six years of obstetric ultrasound experience consecutively scanned the hospitals’ databases for eligible ultrasound cases. The ultrasound scans were performed using the systems Voluson E8 by GE (equipped with a curvilinear 5-2 MHz transducer, Boston, MA, USA) and EpiQ 7 by Philips (equipped with a curvilinear 5-1 MHz transducer, Amsterdam, The Netherlands). The collected data included the ultrasound image, gestational age, and maternal age, as well as the crown–rump length (CRL) measurement.

Upon identifying an eligible case, a single midsagittal plane image was selected, and the measurements were initially acquired using ViewPoint™ 6 Ultrasound Reporting Software (Version 6.12.2) by GE Healthcare by the same sonographer (BA). To test intra- and inter-operator reproducibility, a random sample of cases was exported in a DICOM format to be re-measured using MicroDicom DICOM viewer software (Version 2024-2) [28] by the same sonographer and by another sonographer (BAA) with 5 years of obstetric ultrasound experience. Both sonographers were blinded to the fetal gender and to each other’s measurements, as well as any previous measurements.

### 2.3. Ultrasound Measurements

AGD and GTA measurements were documented from each ultrasound image. AGD was measured by placing a caliper at the inferior base of the genital tubercle and a caliper at the rump location where the distal CRL caliper is placed. For the GTA, the angle was determined by drawing a line along the lower spine and another from the genital tubercle, measuring the resulting angle. To maintain consistency across all measurements, the sonographers involved in the study underwent a training session where they practiced on a random sample of cases. This practice session was critical in ensuring that all sonographers adhered to the same technique and measurement protocols. Although different systems were used, the same standardized measurement protocols were applied across all images, ensuring that the data collected were consistent and reliable.

### 2.4. Statistical Method

Descriptive statistics were computed for all variables, and normality checks were performed to ensure the data met the assumptions for subsequent analyses. Differences between male and female fetuses were assessed using independent samples Student’s t-tests. Point-biserial correlation coefficients were calculated to examine the relationships between fetal gender and continuous variables, such as crown–rump length (CRL), anogenital distance (AGD), and genital tubercle angle (GTA).

To analyze the predictive power, binomial logistic regression was selected due to its suitability for modeling the relationship between a binary dependent variable, such as fetal gender (male or female), and one or more predictor variables. It was designed to model the relationship between fetal gender and the predictors AGD and GTA while controlling CRL. This model allowed us to estimate the odds of the fetus being female with each unit increase in AGD or GTA. Additionally, receiver operating characteristic (ROC) curve analysis was used to assess the diagnostic performance of AGD and GTA, with the area under the curve (AUC) indicating the models’ accuracy. The ROC analysis also helped identify optimal cutoff points for AGD and GTA, based on the highest Youden index, balancing sensitivity and specificity to maximize diagnostic accuracy.

To assess the reproducibility of the measurements, intraclass correlation coefficients (ICCs) were calculated. The interpretation of the results was as follows: 0.00–0.20 indicated ‘poor agreement’; 0.21–0.40 indicated ‘fair agreement’; 0.41–0.60 indicated ‘moderate agreement’; 0.61–0.80 indicated ‘substantial agreement’; and values greater than 0.80 indicated ‘almost perfect agreement’ [29]. All statistical analyses were conducted using Microsoft Excel and SPSS version 29 (IBM Corp., Armonk, NY, USA).

## 3. Results

### 3.1. Descriptive Statistics

**Figure 1:**
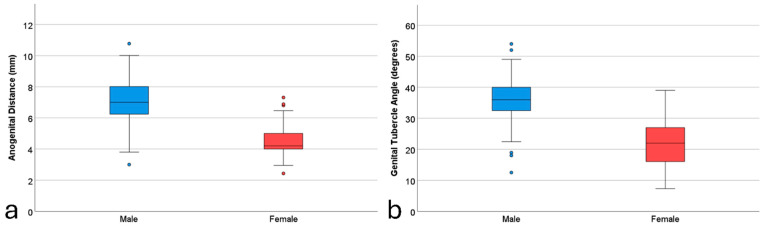
Boxplots of the anogenital distance (**a**) and genital tubercle angle (**b**) measurement differences between male and female fetuses.

**Figure 2:**
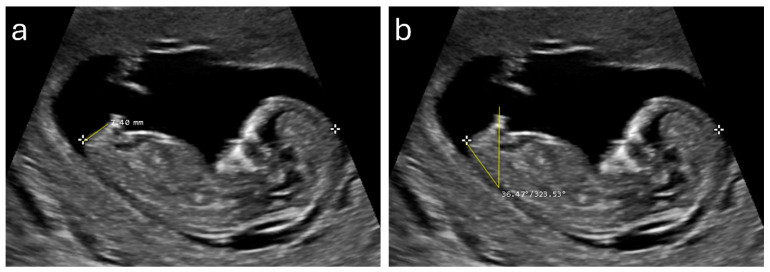
Examples of anogenital distance (**a**) and genital tubercle angle (**b**) measurements from a 12^+4^ week male fetus.

Upon reviewing the records, it was found that approximately two out of every three cases did not have the required midsagittal view acquired. As a result, 324 cases were initially collected for this study. However, 12 cases (3.7%) were excluded due to poor depiction of the genital region, leaving a final total of 312 fetal cases included in the analysis. The descriptive statistics of the main variables are reported in Table 1. The mean AGD [SD, 95% confidence interval (CI)] for male fetuses was 7.16 mm [SD = 1.40, 95% CI = 6.96–7.36], while for female fetuses, it was 4.42 mm [SD = 1.05, 95% CI = 4.23–4.61], demonstrating a mean difference in AGD of 2.74 mm, indicating that the AGD is, on average, 61.2% longer in male fetuses. For GTA, the mean [SD, 95% CI] value for male fetuses was 35.90 degrees [SD = 6.20, 95% CI = 35.01–36.79], compared to 21.57 degrees [SD = 7.29, 95% CI = 20.27–22.87] for female fetuses, showing a mean difference of 14.33 degrees. This indicates that the GTA is, on average, 66.5% larger in male fetuses. The statistical significance of these differences, confirmed by independent samples *t*-tests (*p* < 0.001 for both AGD and GTA), shows the ability of these measurements to distinguish fetal gender. The visual representation in the boxplots (Figure 1) further illustrates the clear difference between male and female fetuses, underscoring the practical applicability of these sonographic markers in clinical settings. Examples of the AGD and GTA measurements are illustrated in Figure 2.

### 3.2. Gender Prediction and Accuracy

Point-biserial correlation between gender at birth and the other independent variables showed statistically significant coefficients with CRL (r = 0.129, *p* = 0.037), AGD (r = 0.792, *p* =< 0.001), and GTA (r = 0.763, *p* =< 0.001); and insignificant coefficients with gestational age (r = −0.090, *p* = 0.123) and maternal age (r = −0.020, *p* = 0.744). Hence, to control for confounding and improve the model accuracy, the CRL was later added to the binomial logistic regression models. 

The first binomial logistic regression model was conducted to predict fetal gender based on AGD and control for CRL, with fetal gender being the dependent variable. The model’s overall fit was statistically significant (χ^2^(2) = 223.213, *p* < 0.001), explaining approximately 69.1% of the variance in fetal gender (Nagelkerke R^2^ = 0.691). The model was able to correctly classify 91.0% of male fetuses and 87.1% of female fetuses, resulting in an overall correct classification rate of 89.4%. When examining the variables individually, AGD was a significant predictor of fetal gender (B = −1.635, SE = 0.175, Wald χ^2^(1) = 87.470, *p* < 0.001). The odds ratio (Exp(B)) for AGD was 0.195, indicating that as AGD increases, the likelihood of the fetus being female decreases. Specifically, for each millimeter increase in AGD, the odds of being female decreased by approximately 80.5%. CRL, while included in the model, was not a statistically significant predictor at the 0.05 level (B = 0.031, SE = 0.018, Wald χ^2^(1) = 3.053, *p* = 0.081).

In the second model, with GTA added instead of AGD, the overall fit was statistically significant (χ^2^(2) = 215.229, *p* < 0.001), explaining 67.4% of the variance in fetal gender (Nagelkerke R^2^ = 0.674). This model had an almost similar accuracy performance to the first model; it was able to correctly classify 92.0% of male fetuses and 82.3% of female fetuses, resulting in an overall correct classification rate of 88.1%. GTA was a significant predictor of fetal gender (B = −0.294, SE = 0.032, Wald χ^2^(1) = 82.170, *p* < 0.001). The odds ratio (Exp(B)) for GTA was 0.745, indicating that as GTA increases, the likelihood of the fetus being female decreases. Specifically, for each degree increase in GTA, the odds of being female decreased by approximately 25.5%. Again, CRL was not a statistically significant predictor (B = −0.013, SE = 0.017, Wald χ^2^(1) = 0.567, *p* = 0.451). 

### 3.3. Optimal Cutoff Points

**Figure 3:**
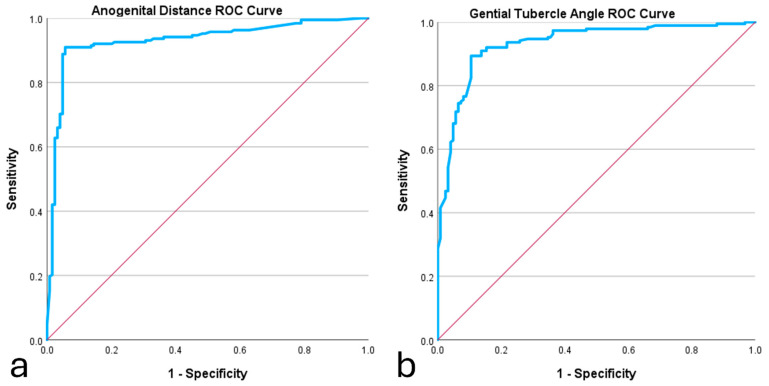
Receiver operating characteristic curves for anogenital distance (**a**) and genital tubercle angle (**b**) measurements.

ROC curve analysis was employed to determine the optimum cutoff points for both measurements. For AGD, the area under the ROC curve (95% CI) was 0.931 (0.899–0.962), reflecting excellent discriminatory ability (*p* < 0.001). The highest Youden Index (0.832) was achieved using the cutoff of 5.74 mm, which had a sensitivity of 94.4% and a specificity of 88.8%. In the case of GTA, the area under the ROC curve (95% CI) was 0.932 (0.904–0.961) with statistically significant discrimination (*p* < 0.001). The cutoff GTA of 28.32 degrees achieved the highest Youden Index (0.767) with 92% sensitivity and 84.7 specificity. In these cases, sensitivity indicates the percentage of male fetuses correctly identified, while specificity indicates the proportion of female fetuses accurately identified. The ROC curves for both measures are represented in Figure 3.

### 3.4. Measurement’s Reproducibility

To evaluate the measurement’s reproducibility, a random subset of 50 cases was re-measured by the same sonographer (BMA) and a secondary sonographer (BDA). The intraclass correlation coefficients for the measurement’s reproducibility within and between the operators were almost perfect (>0.800). Intra-operator reproducibility (95% CI) was 0.938 (0.892–0.965) for AGD and 0.895 (0.815–0.895) for GTA. As for inter-operator reproducibility (95% CI), it was almost perfect at 0.871 (0.776–0.926) for AGD and moderate agreement at 0.695 (0.457–0.828) for GTA.

## 4. Discussion

To our knowledge, this is the first study that compared the predictive performance of AGD and GTA for sex determination during the first trimester. Our results indicate that both markers are highly effective in distinguishing between male and female fetuses, with AGD and GTA demonstrating significant sexual dimorphism. The mean AGD was substantially longer in male fetuses compared to female fetuses, and a similar trend was observed for GTA. The logistic regression models further confirmed the strong predictive power of these markers, with both AGD and GTA significantly contributing to the accurate classification of fetal gender. The ROC curve analyses provided optimal cutoff points for AGD and GTA, which yielded high sensitivity and specificity, underscoring their utility in clinical practice. Additionally, the reproducibility of these measurements was confirmed through high intraclass correlation coefficients, indicating reliable intra- and inter-operator agreement. These findings suggest that AGD and GTA are robust sonographic markers for early fetal sex determination, offering valuable tools for prenatal care and management of gender-specific genetic disorders.

When compared with the previous literature, our mean difference (61%) in AGD between male and female fetuses was higher than that reported in the literature by Alfuraih et al. (15%) in the Saudi population [20], and the 19.5% difference reported by Najdi et al. in a Persian population [17]. However, it was close to the 41.6% and 42.8% differences reported in French and Turkish populations, respectively [23,24]. The study by Alfuraih et al. [20] in a Saudi population had a relatively smaller gestational age compared to the current sample, which may explain the smaller notable difference.

Interestingly, two recent studies published on fetuses of Egyptian ethnicities had almost identical results [21,22]. The two studies published in *The Egyptian Journal of Hospital Medicine* reported on the diagnostic performance of AGD for detecting gender but failed to report on the average reference values. Edris et al. [22] conducted a study on 245 pregnant women and found that the optimal AGD cutoff for predicting fetal gender at 11–12 + 6 weeks was 4.5 mm with an AUC of 0.967–0.988, indicating high diagnostic accuracy. For gestational ages of 13–13 + 6 weeks, a cutoff of 4.9 mm was identified, achieving an AUC of 0.928. The sensitivity and specificity for these cutoffs were notably high, confirming AGD as a reliable marker for early gender prediction. Similarly, Elanwar et al. [21] included 245 pregnant women and reported that the best AGD cutoff value for predicting gender between 11 and 13 weeks was also 4.9 mm, with an AUC of 0.961, a sensitivity of 93.41%, and specificity of 86.49%. 

Furthermore, on the diagnostic performance of AGD, Sipahi et al. [23] demonstrated that AGD measurement could predict fetal sex with 76.7% sensitivity and 95.6% specificity. Another study by Arfi et al. [24] conducted in France reported 87% sensitivity and 89% specificity for AGD in determining fetal sex. Najdi et al. [17] also reported high sensitivity and specificity at 95.0% and 89.6%, respectively. In contrast, Alfuraih et al. [20] indicated that AGD had a sensitivity of 69% and specificity of 60%, suggesting lower discriminatory power compared to other populations. Our findings align closely with those of the former studies, indicating a high sensitivity and specificity of AGD for fetal sex determination. These findings suggest that AGD measurements could accurately predict fetal sex with high accuracy, confirming the robustness of AGD as a reliable marker for early sex determination during the first trimester. 

Despite the high predictive accuracy of AGD, the normative reference ranges varied relatively between the studies, ranging from 3.6 mm to 5.92 mm in females and from 5.10 to 7.16 mm in males [17,20,23,24]. As a result, the suggested optimal cutoff values varied from 4.5 mm to 6.0 mm. The variations in AGD’s reference ranges across different populations highlight the potential influence of genetic and environmental factors on AGD measurements and suggest the need for population-specific reference ranges. Consequently, the optimal cutoff values for sex determination should be race-specific to ensure accuracy.

The racial differences in AGD are also prevalent in older gestational ages as well as infants. In a recent Chinese study, full-term singleton male neonates had 1.99 times longer AGD (23.18 ± 3.17 mm) than female neonates (11.65 ± 1.53 mm) [25]. In contrast, Indian full-term neonates had a larger male/female difference, as the male AGD was >3 times longer at 30.2 ± 3.9 mm compared to 9.3 ± 1.8 mm in female neonates [26]. Older studies also confirm the racial influence on AGD, reporting that white infants have longer AGD than non-white infants [30,31]. Some studies have suggested the potential related factor of maternal hormone variance between races, which may affect fetal hormones as well [32,33]. This characteristic is crucial for researchers to consider, especially when conducting studies with racially diverse samples. 

Our study’s unique contribution lies in comparing AGD against GTA for early fetal sex determination, providing insights into their practical implementation in clinical settings. While both markers demonstrated significant discriminatory power, a significant issue with GTA is the potentially high number of intermediate angles (10–30°), which previous studies have classified as indeterminate results [15,16]. Additionally, it could also be challenging to appreciate the genital tubercle direction, especially in cases when the angle is converging or the tubercle is parallel. Erroneous female sex assignment reports have been documented using the GTA in cases of genital hypospadias where the tubercle could orient downward in male fetuses [34]. This occurs due to the ventral curvature of the penis caused by atresia of the corpus spongiosum close to a malformed hypospadiac opening. The same problem may occur using the GTA technique by misclassifying female fetuses as male in cases of transient hypertrophy of the labia minora [35]. These drawbacks are not present in AGD, where the measurement task is considered to be less challenging and more reliable.

By incorporating AGD and GTA into routine first-trimester ultrasound screenings, clinicians can improve the accuracy of early fetal sex determination, which is crucial for the management of gender-linked genetic disorders. The non-invasive nature of these markers, coupled with their high reproducibility, suggests them as valuable tools in prenatal care. However, the variability in AGD reference ranges across different populations highlights the importance of establishing race-specific reference ranges to ensure diagnostic accuracy and avoid misclassification. Therefore, while AGD shows great promise as a reliable marker, its implementation should be accompanied by efforts to develop standardized guidelines that consider ethnic and population differences. 

A notable strength of our study is the evaluation of inter- and intra-reader reproducibility, a factor seldom reported in previous literature. This assessment significantly enhances the reliability and robustness of our findings, demonstrating the consistency of AGD and GTA measurements. Furthermore, our study utilized samples from two hospitals, adding to the generalizability and robustness of our results. However, our study does have some limitations. Firstly, it was conducted within a single ethnic group, which may limit the generalizability of the findings to other populations. Secondly, the reliance on archived ultrasound images may lead to selection bias, as only cases with available midsagittal plane images were included. This could result in an overestimation of the reproducibility and accuracy of AGD and GTA measurements, as the quality of the images may not reflect the variability encountered in a real-world clinical setting. 

Future research should aim to investigate whether first-trimester AGD measurements can serve as a reliable prenatal indicator of early fetal androgen action postpartum. Longitudinal studies could provide valuable insights into the relationship between AGD measured during the first trimester and various postnatal outcomes related to androgen exposure. This should also be performed in prospective design in which two sonographers independently perform real-time ultrasound measurements on the same patients, ensuring that measurements are taken during live scanning, where optimal visualization of anatomical landmarks can be achieved. Additionally, expanding the research to include diverse populations could help establish race-specific reference ranges and cutoff values, ensuring accurate and culturally sensitive diagnostic practices. Finally, integrating advanced imaging technologies and machine learning algorithms may further enhance the precision and predictive power of AGD and GTA measurements in prenatal care.

## 5. Conclusions

In conclusion, we demonstrated that AGD and GTA are effective sonographic markers for early fetal sex determination during the first trimester. AGD, in particular, showed higher accuracy and fewer potential indeterminate results compared to GTA, highlighting its potential as a superior marker for prenatal sex determination. The study also underscored the importance of considering racial differences in AGD reference ranges and the need for race-specific cutoff values to enhance diagnostic accuracy. Furthermore, our evaluation of inter- and intra-reader reproducibility confirmed the reliability of these measurements, adding robustness to our findings. Given the clinical significance of early and accurate fetal sex determination, future research should focus on validating these markers across diverse populations and exploring their potential as indicators of early fetal androgen action. This study contributes valuable insights to the field of prenatal diagnostics and sets the stage for further advancements in non-invasive fetal sex determination techniques.

## Figures and Tables

**Table 1:** Descriptive statistics of the main fetal and maternal characteristics.

Variable	Male (n = 188)		Female (n = 124)		Overall (n = 312)	
	Mean (SD)	Min–Max	Mean (SD)	Min–Max	Mean (SD)	Min–Max
GA (days)	87.87 (5.62)	77–97	86.24 (5.78)	77–97	87.22 (5.73)	77–97
Maternal age (years)	30.40 (5.19)	16–44	30.17 (4.79)	19–43	30.31 (5.03)	16–44
CRL (mm)	61.25 (10.65)	38.0–85.0	58.59 (10.50)	45.0–83.9	60.19 (10.65)	38.0–85.0
AGD (mm)	7.16 (1.40)	3.0–10.77	4.42 (1.05)	2.43–9.0	6.07 (1.85)	2.43–10.77
GTA (degree)	35.90 (6.20)	8.13–54.0	21.57 (7.29)	7.32–39.0	30.21 (9.67)	7.32–54.0

## Data Availability

Data are available upon reasonable request from the corresponding author.
